# A Novel Signature Based on Angiogenesis-Related Genes Predicts the Prognosis and Immunotherapy Response in HER2-Positive Breast Cancer

**DOI:** 10.7150/jca.94120

**Published:** 2024-07-08

**Authors:** Shuanglong Chen, Weiheng Cui, Jiale Dong, Wenyan Chen, Hongmei Dong, Ruijun Zhao

**Affiliations:** 1 Institute of Precision Cancer Medicine and Pathology, and Department of Pathology, School of Medicine, Jinan University, Guangzhou, China.; 2 School of Nursing and Rehabilitation, Xi'an FANYI University, Xian, China.; 3 Department Medical Oncology, Nanchang People's Hospital, Nanchang, China.; 4 Department of Breast Surgery, Nanchang People's Hospital, Nanchang, China.; 5 Department of General Surgery, The First Affiliated Hospital of Jinan University, Jinan University, Guangzhou, China.

**Keywords:** HER2-positive breast cancer, angiogenesis-related gene signature, immune cell infiltration, immunotherapy, prognosis

## Abstract

**Background:** HER2-positive breast cancer is one of the most prevalent subtypes of breast cancer and represents a significant health concern for women worldwide due to its high morbidity and mortality rates. Recent studies have consistently underscored the pivotal role of angiogenesis in the development and progression of HER2-positive breast cancer. Here, we developed a prognostic signature based on angiogenesis-related genes (ARGs) to categorize HER2-positive breast cancer patients and provide insights into their survival outcomes.

**Methods:** Kaplan-Meier survival curve, time-dependent receiver operating characteristic (ROC) and nomogram were performed to investigate the prognostic performance of the signature. In addition, we comprehensively analyzed the correlation of the prognostic signature with immune cell infiltration, immune checkpoint inhibitors (ICIs) therapy. Finally, Immunohistochemistry (IHC) and immunoblotting were used to investigate XBP1 expression in HER2-positive breast cancer tissues. Colony formation assay was performed to examine cell proliferation of HER2-positive breast cancer cells.

**Results:** The Kaplan-Meier curves and the ROC curves demonstrated that the ARGs had good performance in predicting the prognosis of HER2-positive breast cancer patients. In addition, we observed that the low-risk group was remarkably associated with immune infiltration and better response to ICIs. Further experimental results show that XBP1 is upregulated in human HER2-positive breast cancer, and its knockdown significantly inhibited cell proliferation.

**Conclusions:** Our study demonstrated that the ARGs could serve as a novel biomarker for predicting the prognosis of patients with HER2-positive breast cancer and providing new insights into immunotherapy strategies for these patients.

## Introduction

Based on the 2024 cancer statistics, breast cancer remains a predominant malignancy among women 1. Breast cancer is a highly heterogeneous disease, categorizing into four primary molecular subtypes: Luminal A, Luminal B, HER2-enriched, and basal-like, often referred to as triple-negative breast cancer (TNBC) 2. Of note, approximately 15-20% of breast cancers arise from HER2 gene amplification and excessive protein expression, which is defined as HER2-positive breast cancer, conferring a more aggressive phenotype 3. HER2-positive breast cancer is associated with increased cell proliferation, accelerated angiogenesis and tumor metastasis, reduced apoptosis, and increased resistance to anticancer therapy 4. Currently, trastuzumab combined with adjuvant chemotherapy notably enhances patient survival rates, but it has a limited potential due to drug resistance 5. Thus, the identification of new prognostic biomarkers and therapeutic targets is crucial for enhancing patient outcomes and enabling early diagnosis of breast cancer.

Angiogenesis, which refers to the formation of new capillary blood vessels within the tumor microenvironment, is pivotal in tumor survival, proliferation, and metastasis 6. Breast cancer undergoes angiogenesis during the early stages of growth, invasion, and metastatic spread, making it a reasonable therapeutic target. Notably, angiogenesis has been established as an independent prognostic indicator in breast cancer 7. HER2-positive breast cancer is aggressive with high recurrence rate and mortality, primarily due to increased angiogenesis and a higher risk of metastasis. Studies have shown that various angiogenic growth factors play a significant role in tumor angiogenesis, including vascular endothelial growth factor (VEGF), basic fibroblast growth factor (bFGF), epidermal growth factor (EGF) and angiopoietin, platelet-derived growth factor (PDGF), transforming growth factor β (TGFβ), tumor necrosis factor α (TNFα) 8, 9. Numerous studies have highlighted a positive correlation between elevated levels of angiogenic factors and more advanced stages of breast cancer, leading to a less favorable prognosis 10. Multiple studies have revealed that VEGF is often overexpressed in HER2-positive breast cancer and is linked to reduced disease-free survival (DFS) and overall survival (OS) 11. Both preclinical and initial clinical findings suggest that combining VEGF-targeted therapy with trastuzumab could be advantageous for patients with HER2-positive breast cancer 12. A prior study indicated that elevated serum TGF-α levels forecasted an unfavorable prognostic response to lapatinib and capecitabine in patients with HER2-positive breast cancer 13. Recent studies have uncovered that HER2 not only triggers TGF-β-mediated EMT but also fuels aggressive tendencies in HER2-positive breast cancer 14. The involvement of TGF-β-induced EMT in trastuzumab resistance has been convincingly demonstrated 15. At present, angiogenesis inhibitors are primarily employed in HER2-positive breast cancer patients, and their effectiveness in HER2-negative breast cancer patients warrants further investigation.

A growing body of evidence suggests that angiogenesis may indeed assume a pivotal role in cancer development and progression by influencing the tumor immune microenvironment (TIME) 16, 17. In HER2-positive breast cancer, a robust anti-tumor immune response is linked to improved patient prognosis and therapy response. Reports have highlighted that antiangiogenic therapy can influence the TME through a multifaceted process, and in collaboration with the TME, it modulates the onset and progression of tumors 18, 19. Studies have shown that HER2-targeted resistance was associated with increased levels of TGF-β1 and programmed cell death ligand 1 (PD-L1) and anti-tumor immune response resistance in HER2-positive breast cancer 20. It is notably heartening that antiangiogenic therapies and immunotherapy have found application in a wide spectrum of cancers, including breast cancer 21, 22. Nonetheless, there have been no reports regarding the utilization of an angiogenesis-related genes (ARGs)-based signature as a predictor for HER2-positive breast cancer. Furthermore, the relationship between ARGs with the immunotherapy response in breast cancer remains unclear in HER2-positive breast cancer.

In this study, we established an ARGs using data from The Cancer Genome Atlas (TCGA) cohort and subsequently validated it in Gene Expression Omnibus (GEO) datasets. Collectively, these findings imply that ARGs have emerged as robust prognostic biomarkers and predictive factors for immunotherapy response in HER2-positive breast cancer.

## Materials and Methods

### Sample collection

We collected RNA expression data along with corresponding clinical information from 289 HER2-positive breast cancer patients in the TCGA database. We excluded 11 patients with survival times less than 30 days, resulting in a final cohort of 278 patients who had complete information about the survival status and duration, which was utilized for prognostic analysis. Furthermore, we conducted validation using the Affymetrix Human Genome U133 Plus 2.0 chip (GPL11154) on the GSE202203 dataset, encompassing 369 patients diagnosed with HER2-positive breast cancer and providing comprehensive clinical survival information. The clinicopathological characteristics of the patients in both the training and validation sets are presented in Table S1. A total of 397 ARGs, with relevance scores ≥2.5, were sourced from the GeneCards website (https://www.genecards.org/). However, only 388 of these ARGs were present in the RNA expression data from the TCGA database. Therefore, we ultimately identified and utilized these 388 ARGs for our prognostic analysis.

### Construction of the prognostic ARGs

Differential expression analysis of ARGs between tumor and normal sample was performed by using the “Limma” package in R software. The differentially expressed genes (DEGs) was defined as FDR≤0.05 and absolute log2 fold change (FC)>0.5. Then, univariate Cox regression analyses were conducted to identify DEGs significantly correlated with the OS of HER2-positive breast cancer patients. We identified 8 prognostic ARGs using the R “survival” package with a significance level of *P*<0.05. Moreover, we utilized the “Venn” package in R software to determine the intersection between angiogenesis-related DEGs and prognostic ARGs. The intersection genes as candidate DEGs were used to construct a prognostic ARGs. Subsequently, we employed the least absolute shrinkage and selection operator (LASSO) Cox regression analysis using the “survival” and “glmnet” packages in R software to construct a prognostic model based on ARGs. Finally, we selected 4 ARGs to construct a prognostic signature. The patients' risk score was determined based on gene expression and corresponding Cox regression coefficient as follows: Risk score = 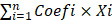 ; Where “i” denotes the number of screened genes, “Coefi” represents the gene correlation coefficient, and “xi” indicates the expression value of each factor, which refer to mRNA levels in this study.

### Validation of the prognostic ARGs

The patients were categorized into two groups based on the median risk score from the TCGA cohort: a high-risk group (n=139) and a low-risk group (n=139). Additionally, patients in the GSE202203 cohort were classified into low-risk and high-risk categories using the same cutoff value. We employed the “survival” package in R software to conduct Kaplan-Meier survival analysis. Time-dependent receiver operating characteristic (ROC) curve and a comparison of ROC curve analysis combined with other clinical features was conducted by “timeROC” and “survival” R package to evaluate the predictive performance of this prognostic signature. Univariate and multivariate Cox regression models were executed to rigorously assess whether the risk score serves as an independent prognostic factor for HER2-positive breast cancer patients.

### Nomogram analysis

To evaluate the 1-year, 2-year, and 3-year OS of patients, we constructed a nomogram incorporating the risk score and key clinical variables for HER2-positive breast cancer patients, including age, clinical stage, T stage, and N stage. A calibration curve was generated to assess the concordance between the predicted and observed OS probabilities.

### Clinical characteristics of prognostic ARGs

The Wilcoxon signed-rank test was employed to assess variations in risk scores among groups with diverse clinicopathological characteristics. The “ggupbr” package was utilized for data analysis and to draw scatter plots. Furthermore, we employed principal component analysis (PCA) using the “Rtsne” package within the R software to assess the effectiveness of prognostic signatures in distinguishing between HER2-positive breast cancer patients with high and low risk, based on gene expression signatures. Simultaneously, we investigated the distribution of various groups through t-distributed stochastic neighbor embedding (t-SNE) analysis, utilizing the “Rtsne” package.

### Immunotherapy combined with immune checkpoint analysis

Immune scores and stromal scores respectively signify the extent of infiltration by distinct immune cells and stromal cells within the tumor tissue. We utilized the “GSEABase” and “GSVA” packages to compute the immune scores of various immune cell types and the activities of immune-related pathways in both high-risk and low-risk groups through single-sample gene set enrichment analysis (ssGSEA). We then assessed the expression levels of immune checkpoint genes in both high-risk and low-risk groups within the TCGA cohort using the “ggpubr” package.

### Functional enrichment and genetic mutation burden analysis

Gene Ontology (GO) and gene set enrichment analysis (GSEA) based on the KEGG database was performed using the “clusterProfiler” R package to determine the differently enriched signaling pathways between high-risk and low-risk group. Furthermore, we gathered genetic mutation data for HER2-positive breast cancer patients from the TCGA database. We employed the “maftools” package in R software to illustrate the genetic mutations in angiogenesis-related differentially expressed genes and visualized them using a waterfall plot.

### Patients

Thirty HER2-positive breast cancer patients underwent surgical treatment at Nanchang People's Hospital from December 2019 to September 2020. Enrollment criteria: (I) simple invasive HER2-positive breast cancer patients, no other systemic diseases (including diabetes, rheumatic diseases, and chronic inflammatory diseases, etc.); (II) preoperative chemotherapy, radiotherapy and other anti-tumor therapy were not performed. Concurrently, comprehensive clinical information was gathered for each patient, including patient age, gender, T stage, N stage, tumor grade and tumor stage. The average age of the patients was 50 years, ranging from 24 to 82 years. The study protocol was subjected to review and approval by the Ethics Committee of Nanchang People's Hospital (Approval number: #K-ky2023049). Written informed consent was obtained from all patients, and all clinical investigations were conducted in accordance with the principles outlined in the Declaration of Helsinki.

### Immunohistochemistry (IHC)

In brief, 4 µm sections were taken from representative breast cancer tumor tissues obtained from formalin-fixed paraffin-embedded specimens. These sections underwent a series of processes, including deparaffinization, rehydration, blocking of endogenous peroxidase activity, and antigen retrieval. We employed the following primary antibody: anti-XBP1 (Cat #ab37152, Abcam, CA, USA). The samples were incubated with the primary antibody overnight at 4°C. Subsequently, the sections were incubated with horseradish peroxidase-conjugated secondary antibodies at room temperature for 1 hour. Hematoxylin was used to counterstain the nuclei. The IHC expression score was determined by multiplying the staining intensity score with the percentage of tumor cells stained. The scoring for the percentage of positively stained cells was as follows: 0, negative; 1, ≤10%; 2, 11-50%; 3, 51-75%; 4, >75%. The staining intensity scores were defined as: 1, weak staining; 2, moderate staining; 3, strong staining.

### Cell culture

We obtained HER2-positive breast cancer cell lines, including BT474 and SKBR-3 cells, from the American Type Culture Collection (ATCC, USA). These cells were cultured in DMEM/F12 medium (Gibco, Invitrogen, Carlsbad, CA), supplemented with 10% fetal bovine serum (FBS), and maintained in a humidified incubator at 37°C with 5% CO_2_.

### Plasmid constructs and transfection

Knockdown of the XBP1 gene in BT474 and SKBR-3 cells was performed using an shRNA lentivirus (GeneChem, Shanghai, China). The plasmid was subsequently transfected into cells by using the Lipofectamine 3000 reagent (Invitrogen, Carlsbad, CA, USA) following the manufacturer's instructions. As a negative control, a scrambled control sequence (shCon) was utilized.

### Colony formation assay

For colony formation assay, BT474 and SKBR-3 cells (1×10^3^ cells per well) with stable XBP1 knock down were seeded into six-well plates. These cells were allowed to adhere and form colonies over a 2-week period. To visualize the colonies, the culture medium was aspirated, and the cells were fixed in methanol for 15 minutes before being stained with 0.1% crystal violet (Sigma) for 20 minutes. Each experiment was conducted in triplicate.

### Immunoblotting

Total proteins were extracted from BT474 and SKBR-3 cells using ice-cold radioimmunoprecipitation assay (RIPA) cell lysis buffer, supplemented with protease inhibitors (Sigma). Equal amounts of protein lysates were separated using 12% sodium dodecyl sulfate-polyacrylamide gel electrophoresis (SDS-PAGE). The isolated proteins were subsequently transferred from the gel onto a polyvinylidene fluoride (PVDF) membrane. These PVDF membranes were blocked with 5% skim milk in Tris-buffered saline with 0.1% Tween-20 (TBST) and incubated for 2 hours at room temperature (RT). Afterward, the membranes were incubated overnight at 4°C with primary antibodies, including anti-XBP1 (Cat #ab37152, Abcam, CA, USA) and anti-β-actin (Cat #4967, Cell Signaling Technology, Beverly, MA, USA). Then, the membranes were exposed to a horseradish enzyme-labeled secondary antibody for 1hour at RT. Lastly, we employed the enhanced chemiluminescence (ECL) kit to detect the protein signal.

### Statistical analysis

All statistical analyses were performed using the R software (version 4.1.3). Data matrix creation and processing were accomplished using the Perl language. When comparing two groups, we utilized the Student's t-test or the Mann-Whitney test for non-normally distributed data, as appropriate. For comparisons involving more than two groups, one-way ANOVA was applied, followed by post-hoc intergroup analysis using the Tukey's test. We employed Kaplan-Meier survival curves to determine cumulative survival, and the log-rank test was utilized to evaluate differences between these curves. Both univariate and multivariate Cox regression analyses were conducted to pinpoint prognostic factors. A two-tailed *P*-value of less than 0.05 was deemed statistically significant.

## Results

### Identification of prognostic angiogenesis-related DEGs in the TCGA cohort

**Figure 1:**
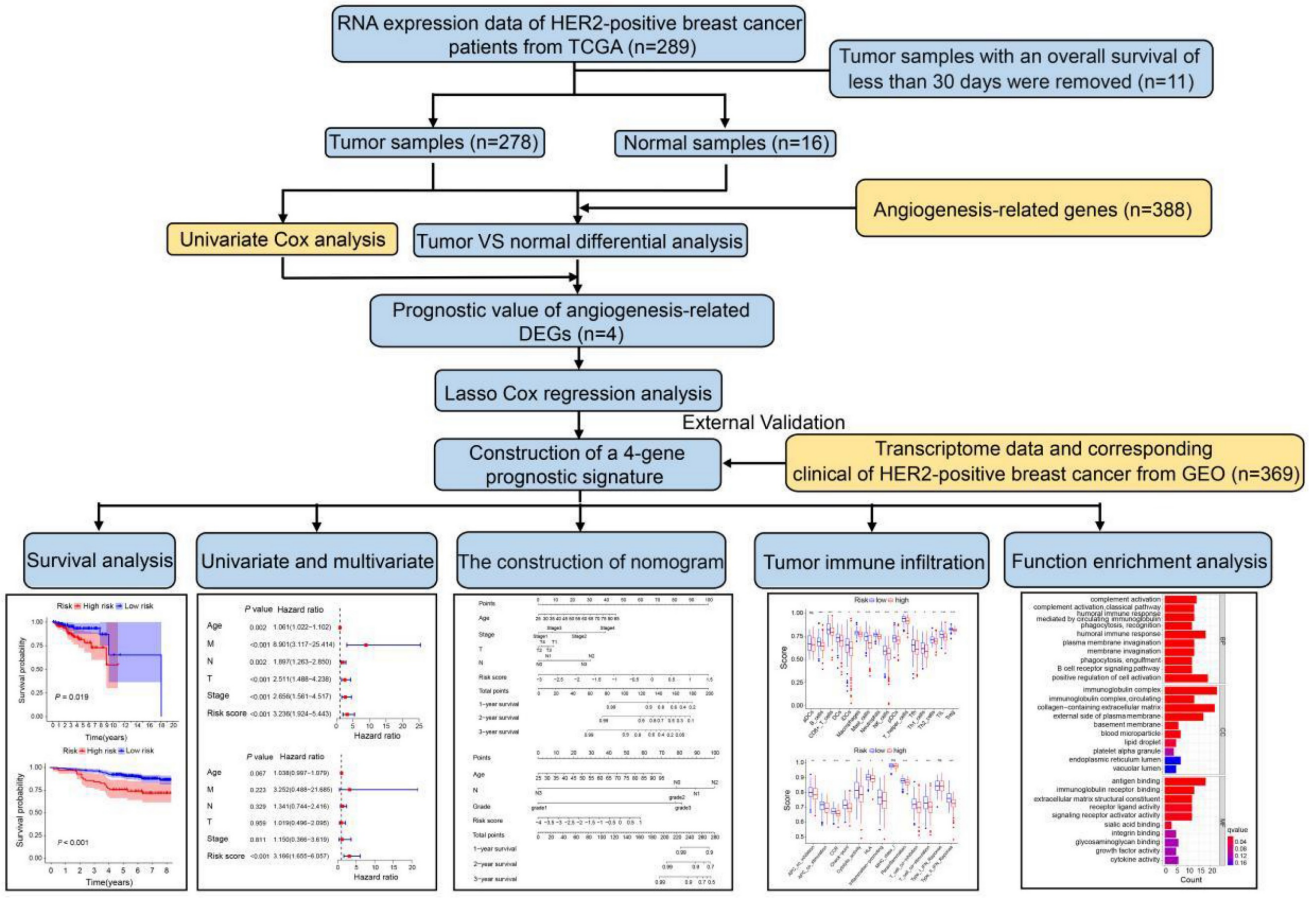
Schematic representation of the study's workflow.

**Figure 2:**
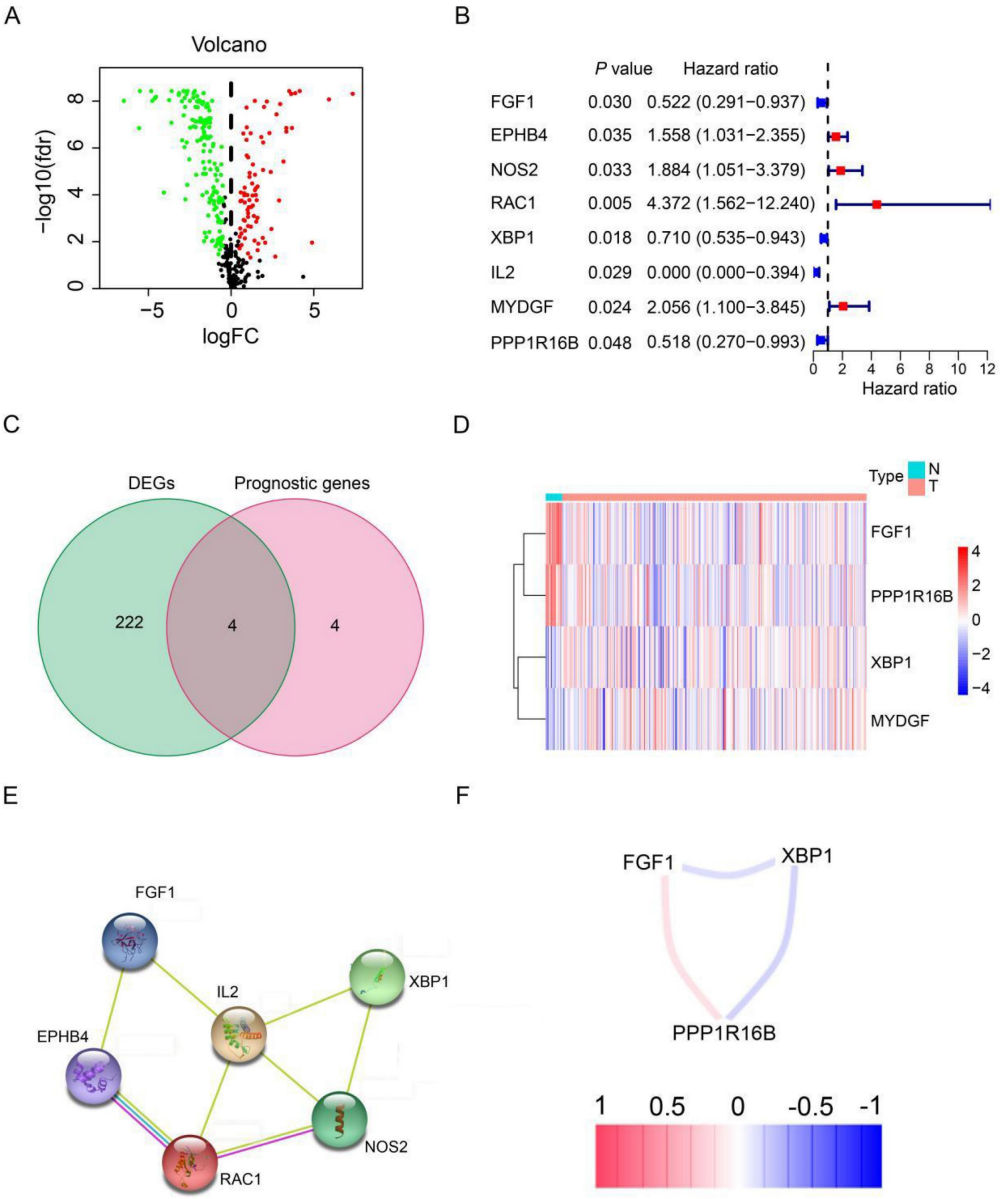
** Expression patterns of angiogenesis-related DEGs in HER2-positive breast cancer.** (**A**) Volcano plot representing DEGs sourced from the TCGA database. (**B**) Forest plot illustrating univariate Cox regression analysis results for the relationship between gene expression and OS. Genes with HR>1 are identified as risk factors for HER2-positive breast cancer, whereas those with HR<1 serve as protective factors. (**C**) A Venn diagram highlighting the overlap between DEGs and prognostic genes. (**D**) Heatmap showcasing the expression patterns of 4 key prognostic DEGs in both tumor and normal tissues. (**E**) The PPI network visualizes interactions among the 8 prognostic genes linked to OS. (**F**) Correlation network for prognostic DEGs, where varied color intensities signify different correlation coefficients.

The study design and workflow are illustrated in Figure 1. To discern genes pertinent to angiogenesis, we compiled 397 ARGs from the GeneCards database. For the training cohort, we chose 278 patients diagnosed with HER2-positive breast cancer from the TCGA database, along with 16 normal patients. Furthermore, our validation cohort incorporated 369 HER2-positive breast cancer patients from the GEO database. Comprehensive clinical details for these patients can be found in Table S1. To delve into the role of ARGs in HER2-positive breast cancer progression, we embarked on a series of analyses. Initially, by contrasting HER2-positive breast cancer patients with normal tissues in the TCGA cohort, we pinpointed 226 DEGs. Out of the identified DEGs, 78 genes were elevated, while 148 exhibited reduced expression (Figure 2A, FDR<0.05, |log2 FC|>0.5). Moving forward, we evaluated the prognostic potential of ARGs in HER2-positive breast cancer. Through a univariate Cox regression analysis, we spotlighted 8 genes with a significant correlation to OS (Figure 2B, *P*<0.05), which we term as prognostic ARGs. Utilizing the Venn package within the R framework, we discerned genes overlapping between DEGs and prognostic ARGs. Consequently, 4 overlapping genes (OGs) were earmarked for further analysis (Figure 2C). The heatmap visualizes the expression patterns of the four prognostic genes across both normal and tumorous tissues (Figure 2D). To elucidate the interconnections among the proteins expressed by the 8 prognostic ARGs, we assembled a protein-protein interaction (PPI) network with the aid of the STRING database. Within these prognostic ARGs, IL-2 stood out as the hub gene, highlighting its probable importance within the interaction network (Figure 2E). Regrettably, no protein interactions emerged between MYDGF and PPP1R16B in relation to the other six genes. Delving deeper, we examined the correlation network diagram of the OGs to shed light on the interrelations among the four PRGs. The varied hues within the diagram signify distinct correlation coefficients (Figure 2F).

### Construction of prognostic ARGs in the TCGA database

**Figure 3:**
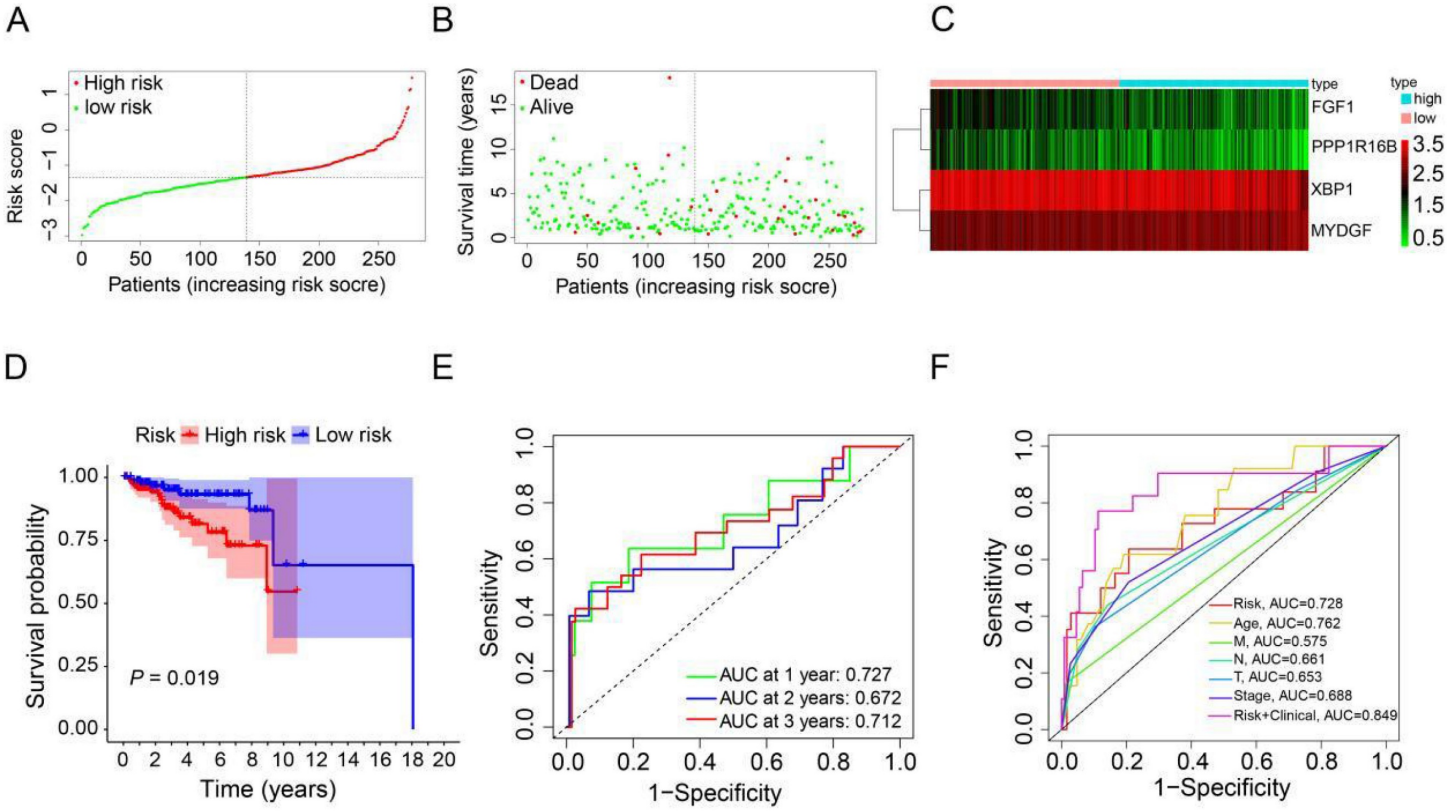
** Construction of a prognostic ARGs using the TCGA database.** (**A**) Distribution of HER2-positive breast cancer patients based on varying risk scores. (**B**) Survival trends of HER2-positive breast cancer patients stratified by risk scores. (**C**) Heatmap showcasing the prognostic ARGs for the training set (TCGA database). (**D**) Kaplan-Meier survival analysis (Log-rank test) comparing the outcomes between high-risk and low-risk groups for HER2-positive breast cancer. (**E**) Time-dependent ROC curves to assess the predictive accuracy of the risk score within the training set using the R package “timeROC”. (**F**) AUC values derived from ROC curves in the training set, comparing the risk model score with clinical parameters.

To derive an optimal prognostic signature, we employed LASSO Cox regression analysis on the expression patterns of the previously mentioned 4 prognostic ARGs. Consequently, we crafted a prognostic signature comprising FGF1, PPP1R16B, XBP1, and MYDGF. We calculated the patients' risk scores using their gene expression data and the corresponding coefficients obtained from LASSO regression. For each patient in the TCGA and GEO cohorts, the risk score was determined using the formula: risk score = [FGF1 expression × (-0.3574)] + [PPP1R16B expression × (-0.4247)] + [XBP1 expression × (-0.2859)] + [MYDGF expression × (0.4376)]. Based on the median value of the risk score in the TCGA cohort, the HER2-positive breast cancer patients were categorized into high-risk (n=139) and low-risk (n=139) groups (Figure 3A). A scatter plot illustrated that with rising risk scores, the probability of earlier death increased for HER2-positive breast cancer patients in the TCGA cohort (Figure 3B). Additionally, a heatmap showcasing the expression patterns of the 4 prognostic ARGs highlighted pronounced disparities between the high-risk and low-risk groups within the TCGA cohort (Figure 3C). To assess the prognostic implications of the risk score for HER2-positive breast cancer patients, we compared survival outcomes between the high-risk and low-risk groups. Kaplan-Meier survival analysis revealed that patients categorized with high-risk scores, based on the prognostic ARGs, experienced notably reduced survival durations compared to their low-risk counterparts in the training group (*P*=0.019, Figure 3D). Subsequently, we employed time-dependent ROC curves to gauge the predictive accuracy of the risk model rooted in the prognostic ARGs for the survival outcomes of HER2-positive breast cancer patients. The area under curve (AUC) values were 0.727 for the first year, 0.672 for the second year, and 0.712 for the third year (Figure 3E). Additionally, the 3-year survival ROC curve was delineated considering factors such as risk, age, stage, T stage, N stage, M stage, and a combination of risk and clinical parameters (Figure 3F). The corresponding AUC values were 0.728, 0.762, 0.688, 0.653, 0.661, 0.575, and 0.849. As highlighted by Figure 3F, the prognostic signature exhibits superior predictive capabilities (AUC=0.849) compared to conventional clinicopathological indicators.

### Validation of prognostic ARGs in the GEO database

**Figure 4:**
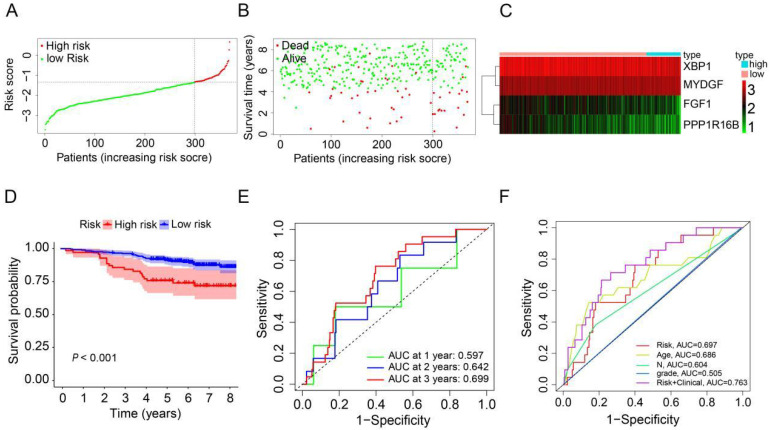
** Validation of the prognostic ARGs in the GEO database.** (**A**) Distribution of HER2-positive breast cancer patients based on varying risk scores. (**B**) Survival analysis of HER2-positive breast cancer patients with different risk scores. (**C**) Heatmap displaying the prognostic ARGs in the validation group (GEO database). (**D**) Kaplan-Meier survival analysis (Log-rank test) comparing outcomes between the high-risk and low-risk groups for HER2-positive breast cancer. (**E**) Time-dependent ROC curves used to validate the prognostic performance of the risk score in the validation group using the R package “timeROC”. (**F**) AUC values for the risk model score compared to clinical features as determined by ROC curves in the validation group.

To evaluate the robustness and generalizability of the prognostic signature, we conducted an external validation utilizing HER2-positive breast cancer samples from the GSE202203 dataset (n=369). Similarly, we computed the risk scores for patients in the validation dataset using the same formula as employed for the TCGA cohort. All HER2-positive breast cancer patients were subsequently stratified into either a high-risk group or a low-risk group, determined by the median risk score (Figure 4A). In the validation cohort, it was evident that patients with high-risk scores generally experienced shorter survival durations compared to their low-risk counterparts (Figure 4B). Furthermore, the heatmap depicting the expression profiles of the 4 prognostic ARGs underscored notable distinctions between the high-risk and low-risk groups in the validation cohort (Figure 4C). The survival curve clearly illustrated that HER2-positive breast cancer patients in the high-risk group faced significantly worse prognoses in comparison to those in the low-risk group (*P*<0.001, Figure 4D). Additionally, the AUC values for predicting OS at 1, 2, and 3 years using the prognostic signature were 0.597, 0.642, and 0.699, respectively (Figure 4E). Additionally, we generated an ROC curve for predicting 3-year survival, considering risk, age, grade, N stage, and a combination of risk with clinical factors (Figure 4F). The corresponding AUC values were 0.697, 0.686, 0.505, 0.604, and 0.763, respectively. In conclusion, these results effectively corroborate the findings from the TCGA cohort, affirming the robustness and generalizability of the prognostic signature in HER2-positive breast cancer.

### Independent prognostic value of the prognostic ARGs in the TCGA and GSE202203 cohorts

**Figure 5:**
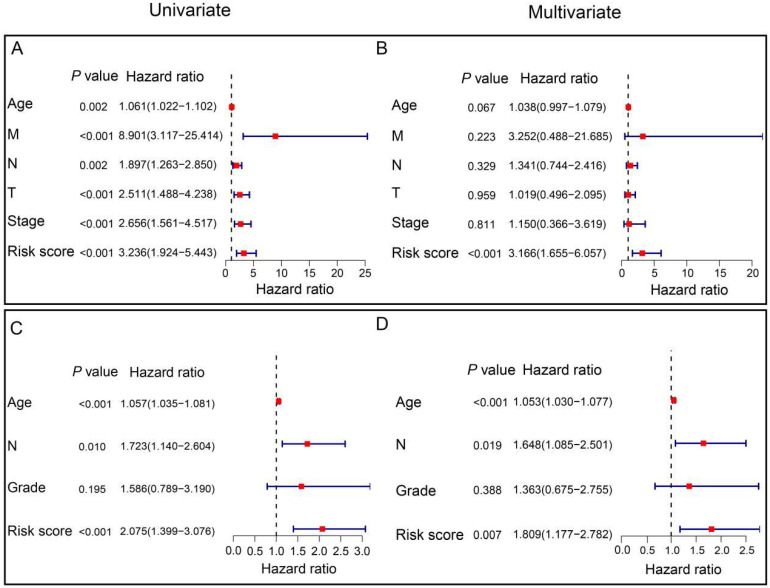
** Independent prognostic value of the signature in HER2-positive breast cancer.** (**A**) Univariate Cox regression analysis illustrating the association between signature-based risk scores and clinicopathologic parameters in the TCGA cohort. (**B**) Multivariate Cox regression analysis examining the relationship between signature-based risk scores and clinicopathologic parameters in the TCGA cohort. (**C**) Univariate Cox regression analysis demonstrating the correlation between signature-based risk scores and clinicopathologic parameters in the GEO cohort. (**D**) Multivariate Cox regression analysis assessing the link between signature-based risk scores and clinicopathologic parameters in the GEO cohort.

To assess the independent prognostic significance of the prognostic ARGs, we conducted univariate and multivariate Cox regression analyses, incorporating clinicopathological parameters from both the TCGA and GSE202203 cohorts. In the TCGA cohort, the univariate Cox analysis unveiled a noteworthy correlation between the risk score and the prognosis of HER2-positive breast cancer patients (HR=3.236, 95% CI: 1.924-5.443, *P*<0.001, Figure 5A). Similarly, within the GSE202203 cohort, the risk score also exhibited a significant association with the prognosis of HER2-positive breast cancer patients (HR=2.075, 95% CI: 1.399-3.076, *P*<0.001, Figure 5C). Consistently, the results from multivariate Cox regression analysis underscored the potential of the risk score as an independent predictor of OS in both the TCGA cohort (HR=3.166, 95% CI: 1.655-6.057, *P*<0.001) and the GSE202203 cohort (HR=1.809, 95% CI: 1.177-2.782, *P*=0.007) (Figure 5B, D). These findings indicate that the risk score based on the prognostic ARGs has independent prognostic value for HER2-positive breast cancer patients.

### Construction of ARGs nomogram

**Figure 6:**
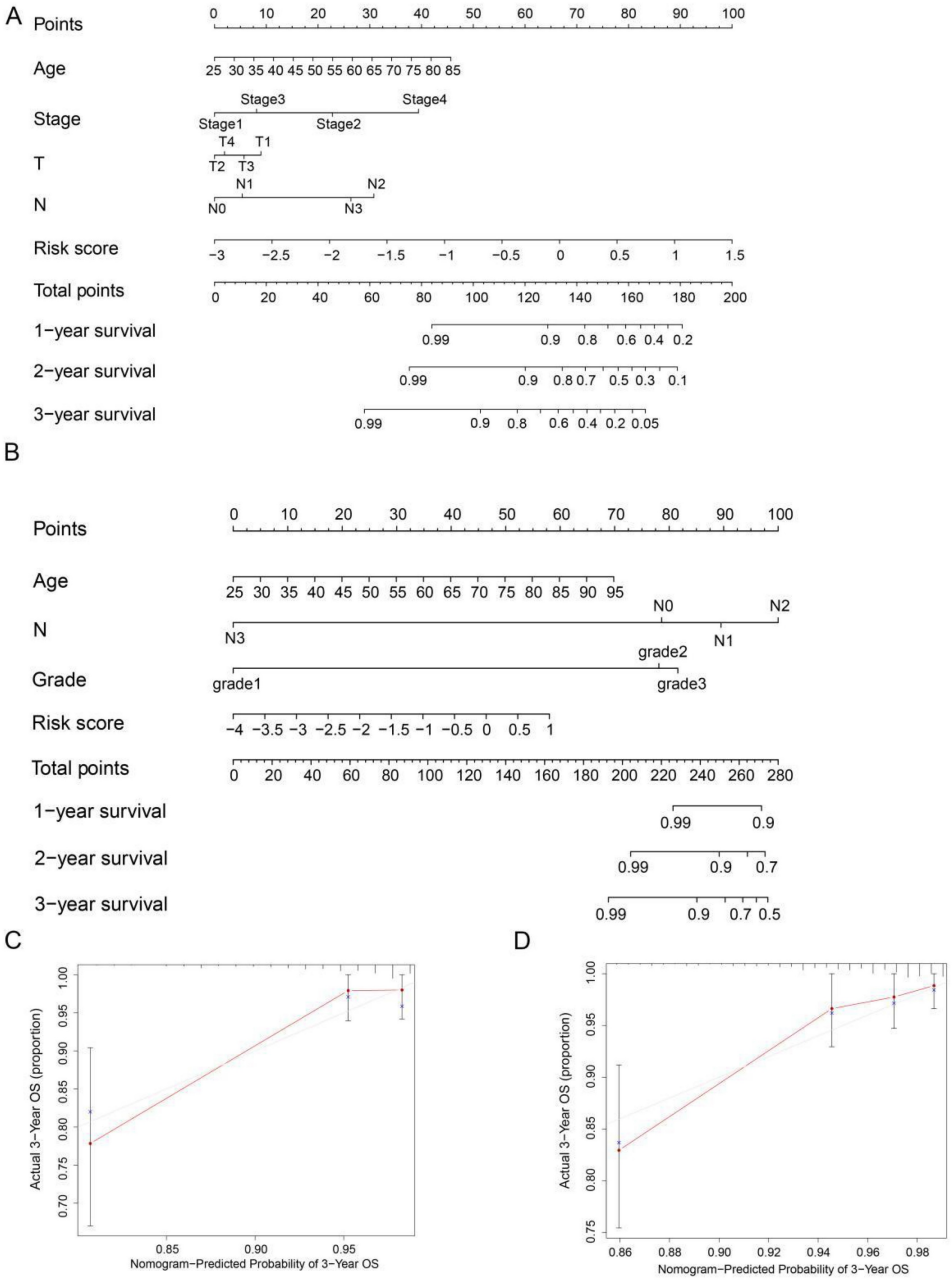
** Nomogram for predicting OS in HER2-positive breast cancer patients.** (**A, B**) Nomograms were depicted using Cox regression analysis to predict OS for HER2-positive breast cancer patients in both the TCGA and GEO cohorts, incorporating the prognostic ARGs and other clinical characteristics. (**C, D**) Calibration analysis illustrating the performance of the prognostic ARGs nomogram in predicting 3-year OS in both the TCGA and GEO cohorts.

To precisely estimate the survival probabilities of HER2-positive breast cancer patients based on their individual characteristics, we developed a nomogram model that incorporates age, stage, T stage, N stage, and risk score, using data from the TCGA cohort. This nomogram model enables the prediction of 1, 2, and 3-year OS probabilities for HER2-positive breast cancer patients (Figure 6A). Similarly, we also constructed a nomogram model in the GSE202203 cohort, incorporating age, grade, N stage, and risk score (Figure 6B). Additionally, to assess the predictive accuracy of the nomogram, we generated calibration plots for the probabilities of 1, 2, and 3-year OS. As a result, the concordance index (C-index) of the nomogram in the TCGA cohort and GSE202203 cohort were 0.839 and 0.748, respectively. In summary, the calibration curves exhibited a favorable agreement between the predicted OS by the nomogram and the actual OS of patients (Figure 6C, D and Figure S1A-D).

### Clinicopathological parameter relevance analysis

To investigate the potential correlation between the prognostic ARGs risk score and clinicopathological variables, we conducted an analysis of the variations in risk scores among distinct subgroups categorized by clinicopathological parameters (age, stage, T stage, N stage, and M stage) within the TCGA cohort. The findings indicated that HER2-positive breast cancer patients aged >60 years exhibited a significantly higher risk score than those aged ≤60 years (Figure S2A, *P*=0.048). Additionally, patients in stage IV had a higher risk score compared to those in stage I (Figure S2B, *P*=0.041). Similarly, the risk score for HER2-positive breast cancer patients with T2, T3 and T4 stage was significantly higher than for those with T1 stage (Figure S2C, *P*<0.05). However, our results did not reveal a statistically significant correlation between the risk score and other clinical parameters, including M stage and N stage (Figure S2D, E, *P*>0.05). In summary, HER2-positive breast cancer patients with higher T stage, older age, and advanced stage appeared to have higher risk scores, indicative of an elevated risk of relapse and poorer prognosis. To further underline the effectiveness of the prognostic ARGs in discerning between high-risk and low-risk groups, we performed PCA and t-SNE. The results showed that the prognostic ARGs successfully stratified high-risk and low-risk groups in both the TCGA and GSE202203 cohorts (Figure S2F-I). To sum up, patients in the high-risk group experienced a notably poorer prognosis in contrast to those in the low-risk group.

### Immune landscape between the low-risk and high-risk HER2-positive breast cancer patients

**Figure 7:**
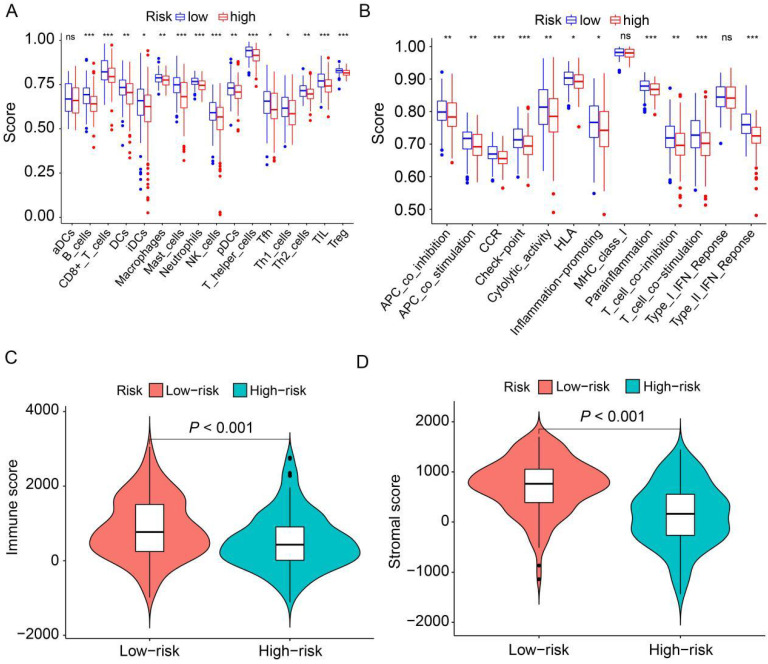
** Analysis of the correlation between prognostic signature and the tumor microenvironment.** (**A**) Box plots depict the scores of 16 immune cells for both high and low-risk groups from TCGA, analyzed using “ssGSEA”. (**B**) Box plots present the scores of 13 immune-related functions for both high and low-risk groups from TCGA, as assessed by “ssGSEA”. (**C**) Illustration of the relationship between risk score and immune score. (**D**) Depiction of the relationship between risk score and stromal score. ns, not significant. *P<0.05; **P<0.01; ***P<0.001 by Student's *t* test.

To delve deeper into the distribution of immune-associated cells and processes between the high-risk and low-risk groups, we applied ssGSEA to compute various immune cell subpopulations and associated enrichment scores for immune functions and pathways within the TCGA cohort. The findings revealed that the high-risk group exhibited lower levels of infiltration by 15 distinct types of immune cells compared to the low-risk group (*P*<0.05, Figure 7A). Furthermore, we observed that 11 immune pathways exhibited reduced activity in the high-risk group compared to the low-risk group (*P*<0.05, Figure 7B). Conversely, MHC class I and type I IFN response did not show statistically significant differences. Additionally, we combined the immune cell score and stromal cell score of HER2-positive breast cancer patients in the TCGA cohort to assess their relationship with the high-risk and low-risk groups. The results demonstrated that both the immune score and stromal score were significantly higher in the low-risk group than in the high-risk group (*P*<0.001, Figure 7C, D). Collectively, these findings suggest that the low-risk group exhibits elevated levels of infiltrating immune cells and increased activity in immune-related pathways compared to the high-risk group.

### Relationship between risk score models and HER2-positive breast cancer patient's mutations

**Figure 8:**
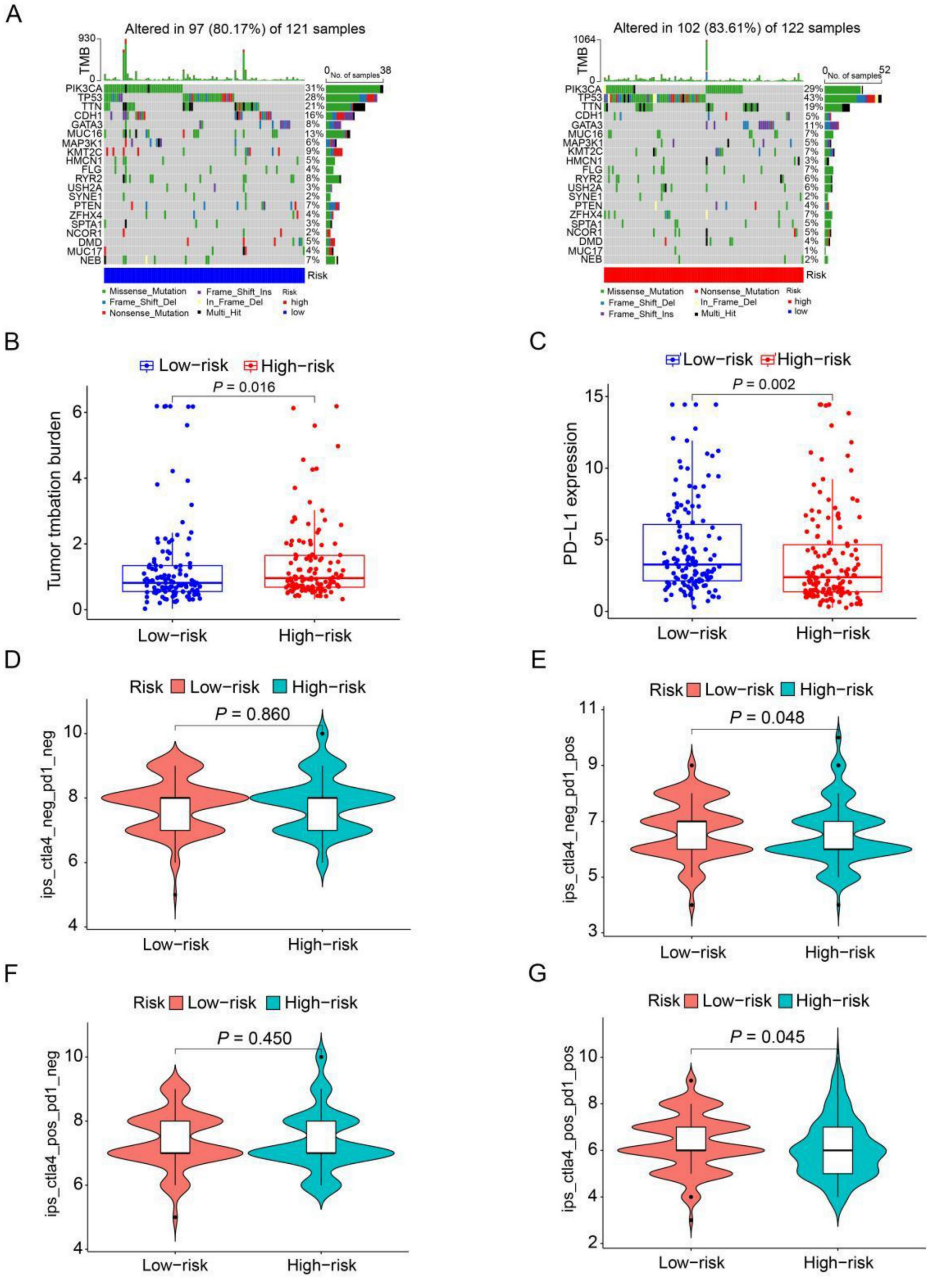
** The mutation profile and TMB among low-risk and high-risk groups.** (**A**) Depiction of the mutation landscape in both low-risk and high-risk cohorts. (**B**) The relationship between the prognostic ARGs and TMB. (**C**) Association between the prognostic ARGs and PD-L1 expression levels. (**D-G**) Comparative analysis of the immunophenoscore (IPS) in the low-risk and high-risk groups, further stratified by both CTLA4 and PD1 markers.

To further analyze the disparities in somatic mutations between the high and low-risk groups, we obtained somatic mutation data from the TCGA database. Subsequently, we explored the relationship between high and low-risk groups and the mutation profile in HER2-positive breast cancer patients utilizing somatic mutation data. In the high-risk group (n=122), the top ten mutated genes included PIK3CA, TP53, TTN, GATA3, MUC16, KMT2C, FLG, ZFHX4, RYR2, and USH2A. Conversely, within the low-risk group (n=121), the top ten mutated genes consisted of PIK3CA, TP53, TTN, CDH1, MUC16, KMT2C, RYR2, GATA3, PTEN, and NEB (Figure 8A). The findings indicate a substantial similarity in the most frequently mutated genes between the low-risk and high-risk groups, underscoring the prevalence of gene mutations in HER2-positive breast cancer patients. Intriguingly, the high-risk group exhibited a notably higher tumor mutation burden (TMB) compared to the low-risk group, indicating a positive correlation between the frequency of somatic mutations and the risk score derived from the prognostic ARGs (*P*=0.016, Figure 8B). Moreover, to shed light on the potential implications of the prognostic ARGs in immunotherapy, we examined the expression of a common immune checkpoint, PD-L1, between the high-risk and low-risk groups within the TCGA cohort. The findings revealed that the expression of PD-L1 was significantly higher in the low-risk group than in the high-risk group (*P*=0.002, Figure 8C), suggesting that patients in the low-risk group may exhibit a more favorable response to immunotherapy compared to those in the high-risk group.

The Immunophenoscore (IPS) serves as a crucial indicator of immune response quality. Furthermore, IPS plays a pivotal role in predicting the responsiveness of HER2-positive breast cancer patients to immune checkpoint inhibitors (ICIs). We consequently delved into the relationship between IPS and the prognostic ARGs risk score. Within the TCGA cohort of HER2-positive breast cancer patients, we employed IPS-CTLA4-neg-PD1-neg, IPS-CTLA4-neg-PD1-pos, IPS-CTLA4-pos-PD1-neg, and IPS-CTLA4-pos-PD1-pos scores to assess the response to ICIs. Notably, IPS-CTLA4-neg-PD1-pos and IPS-CTLA4-pos-PD1-pos scores were significantly higher in the low-risk group (*P*<0.05, Figure 8E, G). Conversely, IPS-CTLA4-neg-PD1-neg and IPS-CTLA4-pos-PD1-neg scores exhibited no statistically significant differences between the high- and low-risk score subgroups (Figure 8D, F). These findings suggest that patients in the low-risk group derive greater benefit from ICI treatment compared to those in the high-risk group.

### GSEA and GO enrichment analyses

To gain insights into the biological processes and pathways influenced by these DEGs, we conducted GO enrichment analysis. The results of the GO enrichment analysis revealed that the enriched biological processes (BP) primarily encompassed positive regulation of cell activation and the humoral immune response. In terms of cellular components (CC), the most enriched category was the collagen-containing extracellular matrix. Moreover, for molecular functions (MF), antigen binding and immunoglobulin receptor binding were significantly enriched (Figure S3A, B). Additionally, to gain deeper insights into the biological functions associated with the prognostic ARGs, we performed GSEA with a cutoff criteria of FDR <25%. The results revealed that oxidative phosphorylation, pyruvate metabolism, alzheimer's disease, huntington's disease, and pyrimidine metabolism were enriched in the high-risk group. In contrast, the low-risk group exhibited enrichment in pathways related to complement and coagulation, ECM receptor cascades, focal adhesion, the JAK-STAT signaling pathway, and leukocyte transendothelial migration (Figure S3C).

### XBP1 is upregulated in human HER2-positive breast cancer

**Figure 9:**
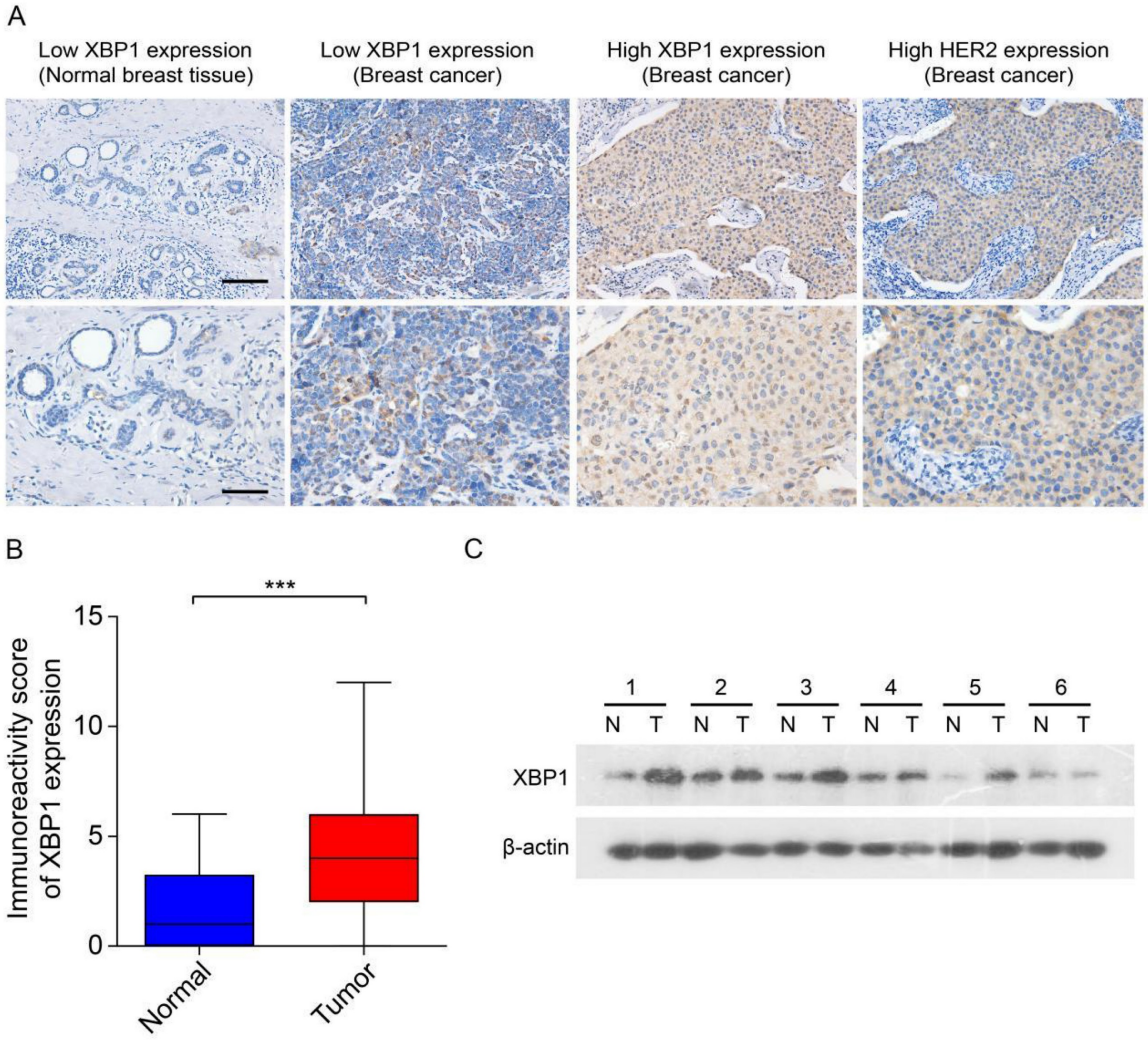
** Expression of XBP1 in human HER2-positive breast cancer. (A)** Representative IHC images depict marked XBP1 overexpression in 30 human HER2-positive samples (T) compared to their corresponding non-tumor adjacent tissues (N). Scale bars, 100 μm. (**B**) A comparative analysis of the immunoreactivity scores for XBP1 between HER2-positive samples (T) and their matched non-tumor adjacent tissues (N). ^***^P<0.001 by Student's *t* test. (**C**) Immunoblotting showcases XBP1 expression levels in 6 human HER2-positive samples (T) and their paired non-tumor adjacent tissues (N). β-actin served as the internal control. Full-length blots (Dotted box) are presented in Figure S4.

Previous data have suggested that XBP1 plays an important role in the development and chemoresistance of HER2-positive breast cancer 23. To explore the role of the prognostic ARGs gene in human HER2-positive breast cancer, we selected XBP1 as a candidate gene for subsequent experimental validation. We assessed the expression of XBP1 through IHC and immunoblotting in 30 randomly selected tumor tissues along with their corresponding adjacent non-tumor tissues obtained from HER2-positive breast cancer patients. The results from IHC indicated a notable increase in XBP1 protein expression within tumor tissues as compared to their matched non-tumor adjacent tissues (Figure 9A, B). Furthermore, the findings from immunoblotting demonstrated that XBP1 expression was upregulated in four out of six cases (66.7%) when compared to their adjacent non-tumor tissues (Figure 9C). In summary, these results collectively confirm the upregulation of XBP1 in human HER2-positive breast cancer.

### Knockdown of XBP1 significantly inhibited cell proliferation

**Figure 10:**
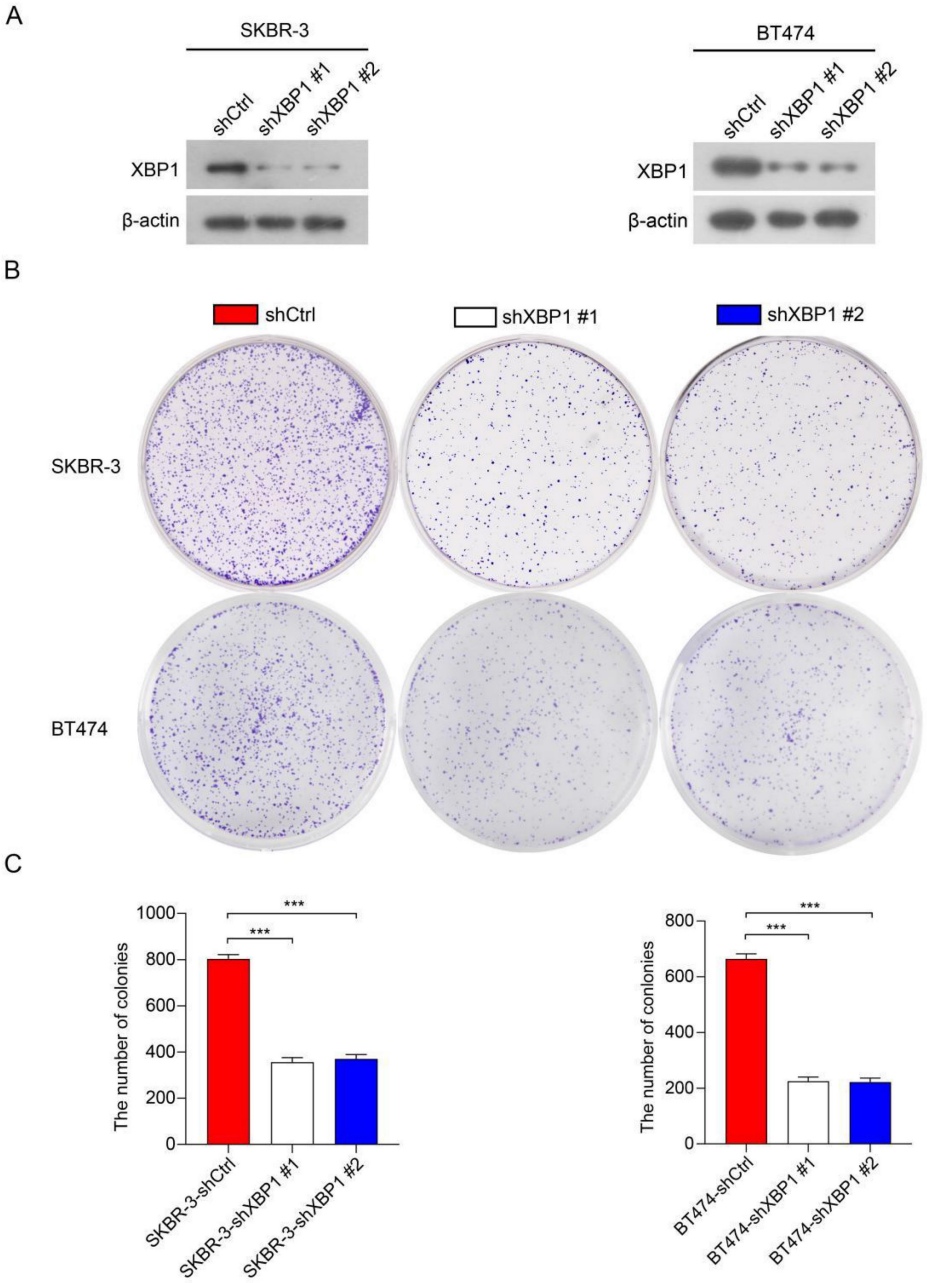
** Knockdown of XBP1 inhibits breast cancer cell proliferation.** (**A**) Immunoblotting analysis of XBP1 protein expression in BT474 and SKBR-3 cells transfected with shXBP1 or control vector. β-actin serves as an internal reference. Full-length blots (Dotted box) are presented in Figure S5. (**B**) Colony formation assays assessed cell proliferation in BT474-shXBP1 and SKBR-3-shXBP1 cells, in comparison with control vector cells. (**C**) A quantitative assessment of the colony numbers derived from the colony formation assays. Data are presented as the average from three independent experiments. Error bars, SEM; ^***^P<0.001 by one-way ANOVA with *post hoc* intergroup comparisons.

To further investigate the roles of XBP1 in HER2-positive breast cancer cells, we employed two distinct short hairpin RNAs (shRNAs), namely shXBP1 #1 and shXBP1 #2, as well as a non-targeting control shRNA (shCtrl) to silence XBP1 in BT474 and SKBR-3 cells. Immunoblotting assays clearly demonstrated a significant reduction in XBP1 protein expression in BT474-shXBP1 and SKBR-3-shXBP1 cells in comparison to those transfected with the control shRNA (Figure 10A). We conducted a colony formation assay to assess the impact of XBP1 on the proliferation of BT474 and SKBR-3 cells. The results revealed that knocking down XBP1 considerably diminished the number of cell colonies in comparison to the control group (Figure 10B, C). Collectively, these findings suggest that XBP1 plays a pivotal role in promoting proliferation in HER2-positive breast cancer cells.

## Discussion

Previous reports have highlighted the significant heterogeneity in tumor cells and the immune microenvironment of HER2-positive breast cancer 24. Presently, the available treatment options for patients with HER2-positive breast cancer remain notably limited. These patients face a challenging prognosis and reduced survival rates following treatment, primarily attributed to the tumor's ability to thrive within an immune microenvironment conducive to its growth, alongside its heightened proliferation and metastatic potential 25, 26. HER2-positive breast cancer represents a highly heterogeneous disease with a complex etiology, and angiogenesis plays a pivotal role in its development and progression 27. Hence, there exists a compelling need for HER2-positive breast cancer patients to investigate an early angiogenesis-related prognostic biomarker that can facilitate risk stratification and targeted therapy. A previous study has unveiled that an ARGs can function as a prognostic biomarker capable of predicting outcomes in breast cancer 28. Nevertheless, the precise roles of angiogenesis and immune microenvironment infiltration in HER2-positive breast cancer remain incompletely elucidated. In our study, we embarked on an investigation into the potential utility of ARGs as a novel biomarker for prognostic assessment in HER2-positive breast cancer.

In this study, we formulated a prognostic ARGs comprising four genes based on the TCGA cohort. Subsequently, we validated its prognostic significance in HER2-positive breast cancer patients, utilizing the GSE202203 database. Our survival analysis unveiled a robust correlation between the risk score derived from the ARGs and OS among HER2-positive breast cancer patients. Univariate and multivariate Cox analyses revealed that the risk score derived from the ARGs could serve as an independent predictor of OS in these patients. To comprehensively compare the performance of our ARGs prognostic signature in predicting OS among HER2-positive breast cancer patients, we calculated the AUC values and C-index of three previously published signatures in the TCGA cohort 29-31. As a result, the AUC value of two signatures (Jia L and Sha R signatures) for 1, 2 and 3 years were lower than those of our ARGs prognostic signature (Figure S6A, B and D), while Liu Q signature had comparable AUC values (Figure S6C). Additionally, our ARGs prognostic signature achieved a C-index of 0.689, ranking second (Figure S6E). These results demonstrated that our model has comparable or even superior clinical efficacy in predicting the prognosis of HER2-positive breast cancer, highlighting the excellent predictive power and potential for broader application.

Additionally, we developed a nomogram based on the ARGs for predicting OS in HER2-positive breast cancer patients, both in the TCGA cohort and the GEO cohort. The nomogram yielded promising results, exhibiting C-indexes of 0.839 and 0.748 in the TCGA and GEO databases, respectively, underscoring its robust prognostic capability. Calibration analysis and decision curve analysis (DCA) further affirmed the clinical utility of the nomogram. These discoveries emphasize the significance of the ARGs in predicting OS among HER2-positive breast cancer patients. The nomogram based on this signature offers a valuable and practical tool for individualized prognosis assessment in clinical practice. The prognostic model presented in this study incorporates four ARGs: FGF1, PPP1R16B, XBP1, and MYDGF. These genes have been the subject of extensive research within the context of breast cancer, and their pivotal roles in its pathogenesis have been established. Fibroblast growth factor 1 (FGF1) is a multifaceted regulator involved in various biological and physiological processes. It has been shown to promote fibroblast mitosis, stimulate blood vessel formation, and facilitate mesoderm cell growth. FGF1 is also recognized for its significant role in wound healing and limb regeneration. Furthermore, several studies have reported a noteworthy association between FGF1 expression and the risk of breast cancer 32, 33. These findings underscore the pivotal role of FGF1 in the development and progression of breast cancer. Protein phosphatase 1, regulatory (inhibitor) subunit 16B (PPP1R16B) is predominantly situated in the plasma membrane and plays a critical role in intracellular signal transduction. Elevated levels of PPP1R16B transcripts have been linked to advanced disease stages and unfavorable prognoses across various cancer types, including breast, colon, esophageal, lung, and prostate cancers. Moreover, the methylation status of the PPP1R16B locus has been identified as a specific diagnostic biomarker for colorectal cancer 34. Furthermore, PPP1R16B has been implicated in facilitating metastasis in pancreatic cancer 35, emphasizing its significant role in cancer progression. Interestingly, previous research has demonstrated that PPP1R16B protein expression is upregulated across all breast cancer subtypes including HER2 positive breast cancer; however, its prognostic role was observed exclusively in the HER2-negative group and not in HER2-positive group 36. Our findings suggest that ARGs consisting of PPP1R16B, FGF1, XBP1 and MYDGF were associated with prognosis in patients with HER2-positive breast cancer. The reason for this difference may be due to the small sample size of the previous study (only 2 out of 32 HER2 positive breast cancer patients with negative PPP1R16B expression), resulting in a bias in the results 36. Myeloid-derived growth factor (MYDGF) (also known as IL25), which encodes the Myeloid Derived Growth Factor protein, is a recently discovered secreted growth factor. Recent studies have compellingly demonstrated that MYDGF plays a pivotal role in the context of acute myocardial infarction (AMI) and manifests as a crucial factor in exerting protective effects during its pathogenesis 37. Furthermore, MYDGF has also been linked to the progression of hepatocellular carcinoma 38. Importantly, MYDGF promotes tumor cell proliferation and is associated with poor breast cancer prognosis, while blocking MYDGF inhibits breast cancer metastasis 39-41. Nonetheless, the precise involvement of MYDGF in the progression of HER2-positive breast cancer and its impact on the tumor microenvironment remains largely unexplored. X-box binding protein 1 (XBP1) was originally discovered through cloning and characterized as a distinctive basic-region leucine zipper (bZIP) transcription factor, primarily involved in the regulation of human MHC class II gene expression. The transcriptional activity mediated by XBP1 aids cellular adaptation to endoplasmic reticulum (ER) stress and promotes tumor progression, concurrently dampening the anti-tumor immune response 42. Previous data have suggested that loss of XBP1 disrupts angiogenesis, inhibits cell proliferation, significantly inhibits breast cancer growth, and promotes sensitization to chemotherapy in a mouse model of HER2 positive breast cancer 23. While its involvement in shaping the tumor microenvironment and its influence on the progression of HER2-positive breast cancer have not been comprehensively explored. In order to furnish additional evidence regarding the expression of XBP1 in HER2-positive breast cancer tissues, we conducted an examination of XBP1 expression in 30 tumor tissues and their corresponding adjacent non-tumor tissues derived from patients with HER2-positive breast cancer, employing both IHC and immunoblotting assay. Our results revealed a significant upregulation of XBP1 in human HER2-positive breast cancer. Furthermore, upon silencing XBP1 in two HER2-positive cell lines, we observed a notable reduction in the proliferation capabilities of HER2-positive breast cancer cells. This study is pioneering in its approach by utilizing a combination of four genes as prognostic indicators for HER2-positive breast cancer patients, offering fresh perspectives on the prognostic value and potential mechanisms underlying this particular subtype of breast cancer.

Recent years have seen an increasing body of evidence highlighting the association between genetic alterations and the responsiveness to immunotherapy in a variety of cancer types, including breast cancer 43, 44. In this study, we delved into the assessment of TMB in both high-risk and low-risk groups, recognizing TMB as a predictive marker for immunotherapy responsiveness. Interestingly, we observed a significant decrease in TMB with elevated risk scores in HER2-positive breast cancer patients. Notably, the top five mutated genes in both the high-risk and low-risk groups were PIK3CA, TP53, TTN, GATA3, and MUC16, implying a degree of similarity in mutation profiles between the two groups (Figure 8A). However, it's imperative to highlight that the TP53 mutation burden in the high-risk group was markedly elevated compared to the low-risk group. Recent research has pinpointed the TP53 mutation as a promising biomarker for HER2-positive breast cancer 45. Moreover, the extent of tumor mutations was considerably greater in high-risk HER2-positive breast cancer patients than in their low-risk counterparts (Figure 8B). This discovery poses significant queries and necessitates in-depth deliberation, as it could shed light on the intricate relationship between gene mutations and immunotherapy in breast cancer.

To our knowledge, this is the first instance of using ARGs to predict outcomes in HER2-positive breast cancer. Angiogenesis plays a critical role in the growth and metastatic spread of breast cancer, and there is increasing interest in the application of antiangiogenic drugs in current breast cancer therapy research. While targeting angiogenesis inhibition holds promise as a therapeutic strategy, the clinical response of currently approved inhibitors has been hampered by notable drug-related side effects and the rapid emergence of resistance mechanisms 46-50. Bevacizumab, a widely recognized antiangiogenic agent, functions by specifically targeting VEGF and inhibiting its interaction with VEGF receptors. It is important to note, however, that bevacizumab does not impact vasculogenic mimicry (VM), which can potentially contribute to drug resistance in cancer cells 51, 52. Sorafenib, a multi-target tyrosine kinase inhibitor, exerts its therapeutic effects by targeting not only VEGF receptors but also other kinases that are involved in tumor growth, such as PDGF receptors. However, when administered as a monotherapy, sorafenib has not demonstrated substantial efficacy in significantly prolonging progression-free survival (PFS) 53, 54. In this study, we found that patients in the high-risk group stratified based on ARGs had significantly shorter survival times than those in the low-risk group (*P*=0.019, Figure 3D). These findings suggest that the implementation of novel targeted therapies aimed at key angiogenic factors, including FGF1, XBP1, PPP1R16B, and MYDGF, in combination with conventional chemoradiotherapy and targeted therapy, has the potential to significantly improve therapeutic outcomes. As the utilization of immunotherapy continues to rise in breast cancer treatment, PD-L1 expression has emerged as a widely adopted biomarker for tumor immune checkpoint therapy. Additionally, angiogenesis inhibitors can reduce immunosuppression in the tumor microenvironment and enhance the antitumor efficacy of immune checkpoint inhibitors by promoting T-cell activation, thus providing a strong rationale for combination trials 55. Our study findings indicate that the low-risk group exhibited significantly higher levels of PD-L1 expression in tumor tissues compared to the high-risk group. This observation suggests a stronger potential for PD-L1 blockade to be effective in the low-risk group, while indicating a potential lack of benefit from PD-L1 blockade in the high-risk group, potentially leading to immunotherapy resistance (*P*=0.002, Figure 8C).

Furthermore, to validate the consistency of clinical information within the datasets utilized, we conducted a comparative analysis of clinical parameters among patients from three cohorts: the TCGA cohort, the GEO cohort, and our in-house cohort. The results indicated that there were no significant differences in the distribution of age, gender, tumor stage, tumor grade, and T stage among patients in our cohort, the TCGA cohort, and the GEO cohort (Table S1, *P*>0.05). Notably, although there are differences in the distribution of N stage between our cohort and the GEO cohort, as well as between the TCGA and GEO cohorts (Table S1, *P*<0.05), the number of patients in N0 stage exceeds those in N1-N3 stages in all cohorts, suggesting a general consistency in the distribution of N stages. Therefore, we believe that the patients in our cohort, the TCGA cohort, and the GEO cohort demonstrate considerable compatibility, allowing for robust comparative analyses. However, there are several limitations in this study as well. First, the data for this study were derived from public databases, the current study constituted a small sample size, and further validation studies with lager clinical patient samples are necessary. Secondly, the potential molecular mechanism by which the ARGs influences immune cell infiltration levels and immune checkpoint molecules in cancers requires deeper exploration, either through *in vivo* or *in vitro* experiments.

## Conclusions

In conclusion, we have developed a novel prognostic model founded on four ARGs, demonstrating its potential in forecasting the prognosis of patients with HER2-positive breast cancer. Additionally, our findings reveal notable disparities in the tumor microenvironment and response to immunotherapy between the high-risk and low-risk patient groups. These insights could prove invaluable for exploring the potential benefits of immunotherapy in treating HER2-positive breast cancer patients.

## Supplementary Material

Supplementary figures and table.
