# Supplementary material for: A Novel Signature Based on Angiogenesis-Related Genes Predicts the Prognosis and Immunotherapy Response in HER2-Positive Breast Cancer
Source: J Cancer. 2024 Jul 8;15(14):4731–48. doi: 10.7150/jca.94120 (PMC11242330; doi:10.7150/jca.94120)
Supplement: Supplementary file 1 — Supplementary figures and table. [file jcav15p4731s1.pdf]

Supplementary information

Supplementary Table

Supplementary Table 1 Clinical and pathological characteristics of patients.

	In-house cohort	TCGA cohort	GEO cohort	*P* value[a]	*P* value[b]	*P* value[c]
No. of patients	30	278	369			
Age				0.729	0.780	0.865
≥60	16	139	187			
<60	14	139	182			
unknow	0	0	0			
Sex				0.508		
Female	30	274	NA			
Male	0	4	NA			
unknow	0	0	369			
Stage				0.575		
Stage I	7	47	NA			
Stage II	15	161	NA			
Stage III	8	60	NA			
Stage IV	0	7	NA			
unknown	0	3	369			
Grade					0.309	
G1	1	NA	3			
G2	5	NA	85			
G3	24	NA	270			
unknown	0	278	11			
T stage				0.111		
T1	7	67	NA			
T2	16	174	NA			
T3	7	27	NA			
T4	0	10	NA			
unknown	0	0	369			
N stage						
N0	13	131	293	0.084	0.000	0.000
N1	10	91	62			
N2	7	28	9			
N3	0	23	1			
unknown	0	5	4			
M stage						
M0	0	233	NA			
M1	0	8	NA			
unknown	0	37	369			

[a]χ^2 test was used for comparing cancer patients in-house cohort and TCGA cohort.
[b]χ^2 test was used for comparing cancer patients in-house cohort and GEO cohort.
[c]χ^2 test was used for comparing cancer patients TCGA cohort and GEO cohort.

Supplementary Figures and Figure legends

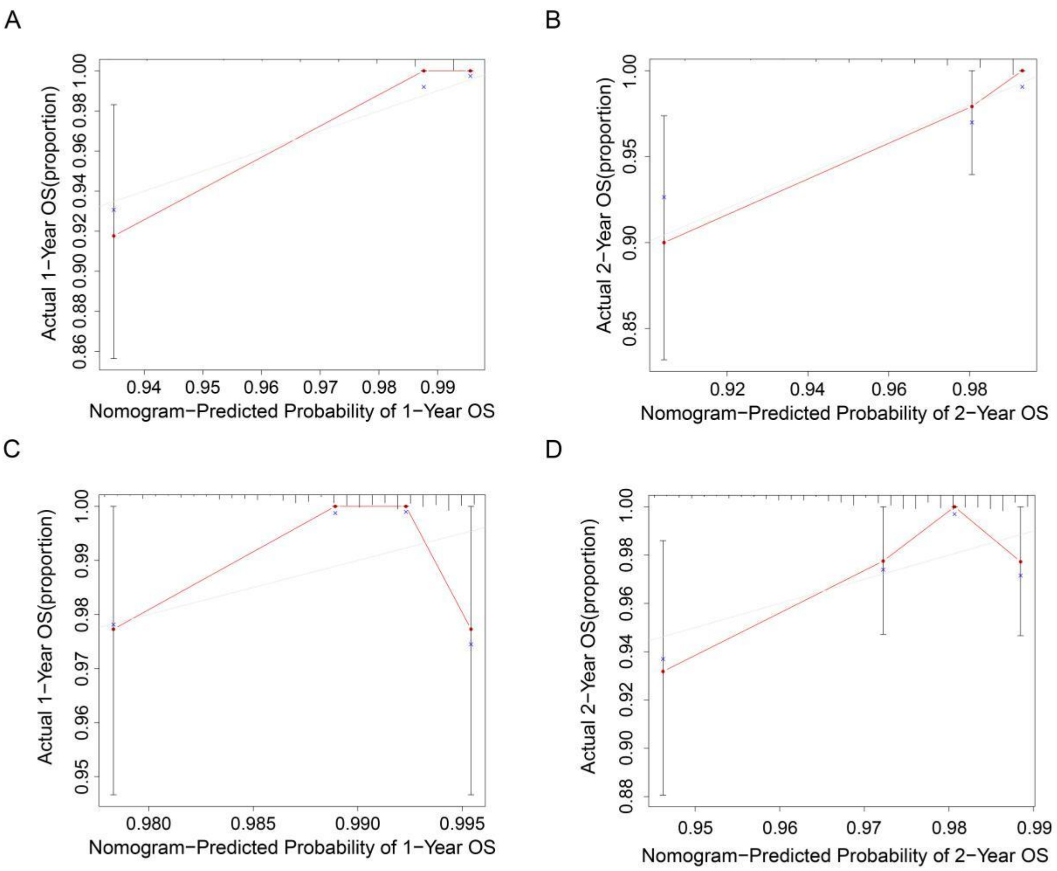

Supplementary Figure 1. Calibration analysis of the prognostic ARGs nomogram. (**A, B**) Calibration analysis illustrating the performance of the prognostic ARGs nomogram in predicting 1 and 2-year OS in the TCGA cohort, respectively. (**C, D**) Calibration analysis illustrating the performance of the prognostic ARGs nomogram in predicting 1 and 2-year OS in the GSE202203 cohort, respectively.

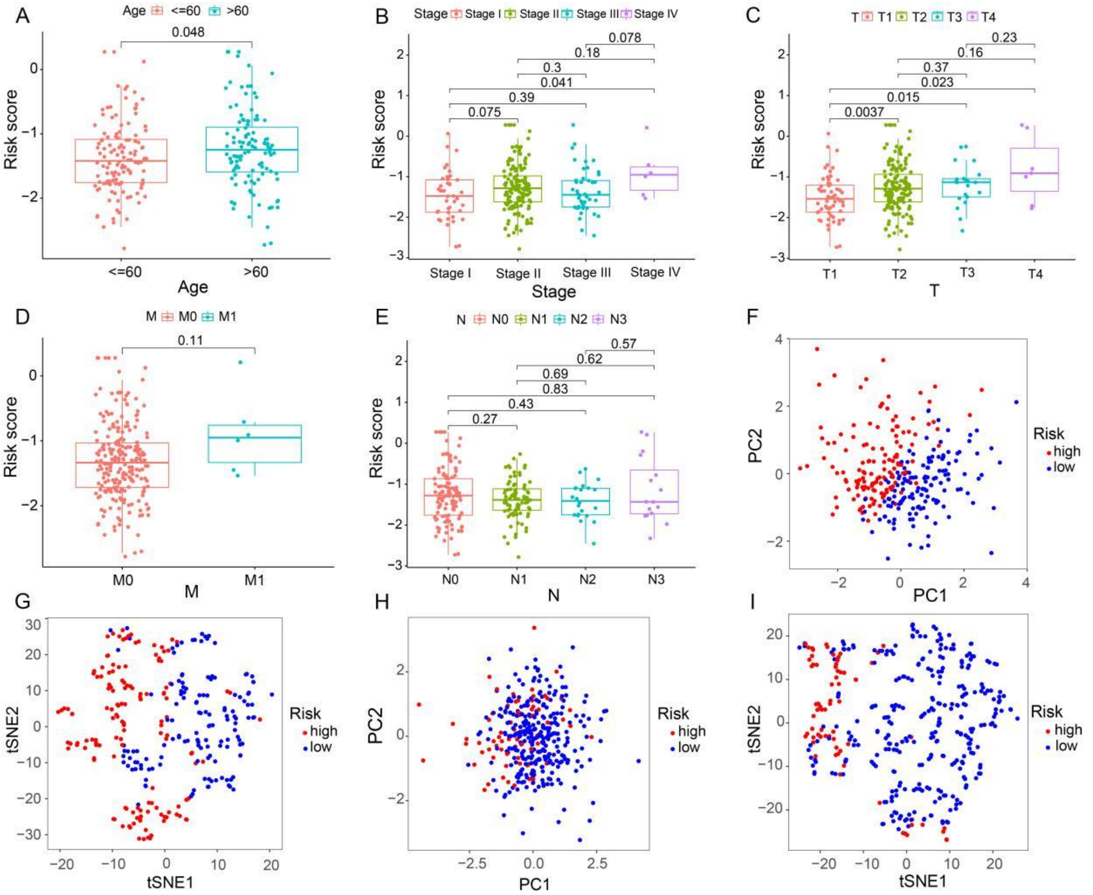

Supplementary Figure 2. Clinical correlation analysis of prognostic signature. Scatter plots illustrate the relationships between risk scores and various clinical pathological features: Age (**A**), Stage (**B**), T stage (**C**), M stage (**D**), and N stage (**E**). (**F**) and (**G**) PCA and t-SNE analysis plot in the TCGA cohort. (**H**) and (**I**) PCA and t-SNE analysis plot in the GEO cohort.

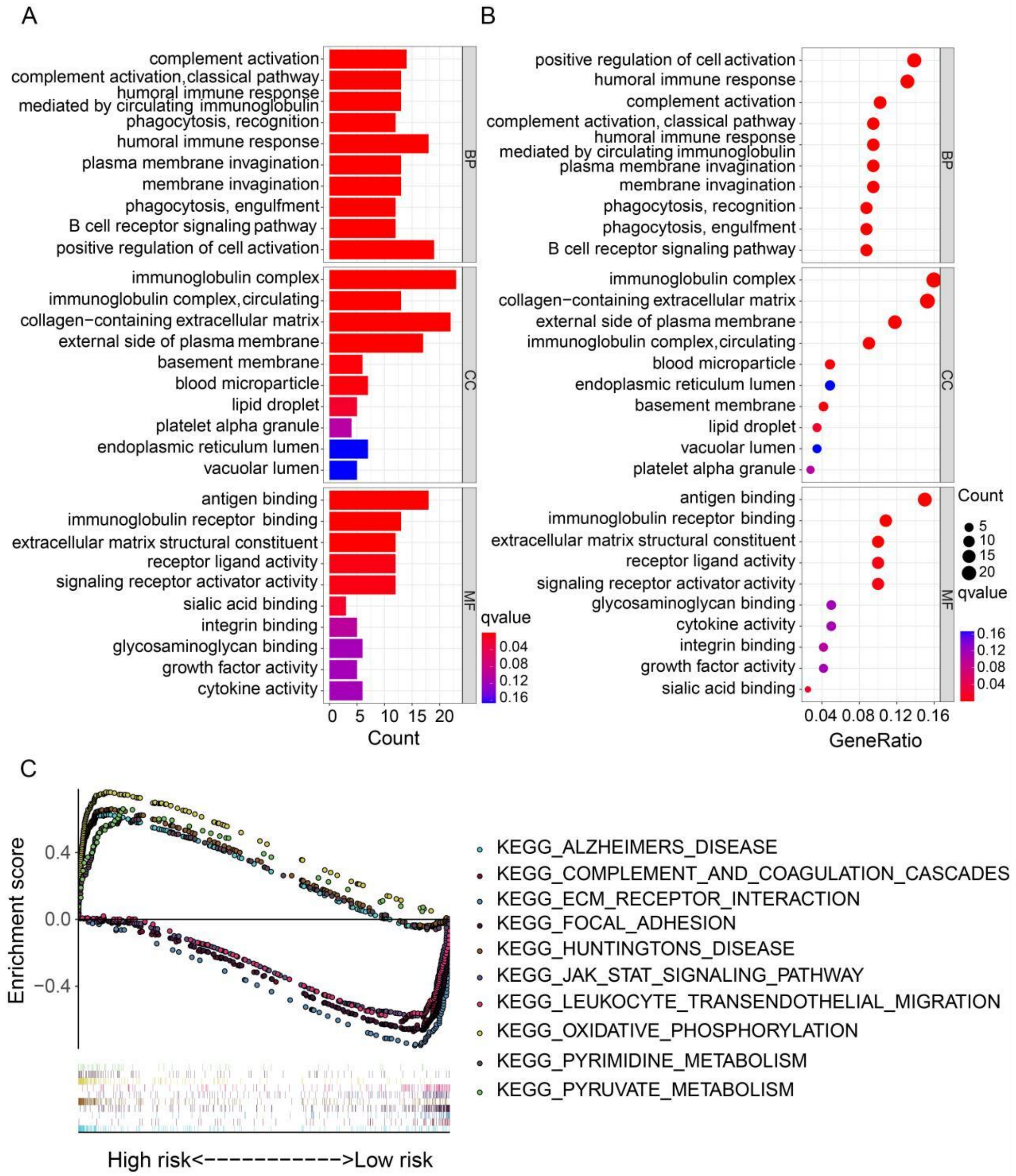

Supplementary Figure 3. Representative results of Gene Ontology (GO) enrichment analyses in TCGA. (**A**, **B**) The results of GO biological process (BP) enrichment, GO cellular component (CC) enrichment, and GO molecular function (MF) enrichment of DEGs. (**C**) Gene set enrichment analysis between high-risk and low-risk groups. The c2.cp.kegg.v7.0.symbols.gmt gene set was used as reference. The maximum and minimum sizes for gene sets were set at 325 and 15, respectively. FDR is the adjusted *P*-value after multiple hypothesis testing, FDR<25% (Benjamini-Hochberg) was used as a cutoff to identify significant gene sets.

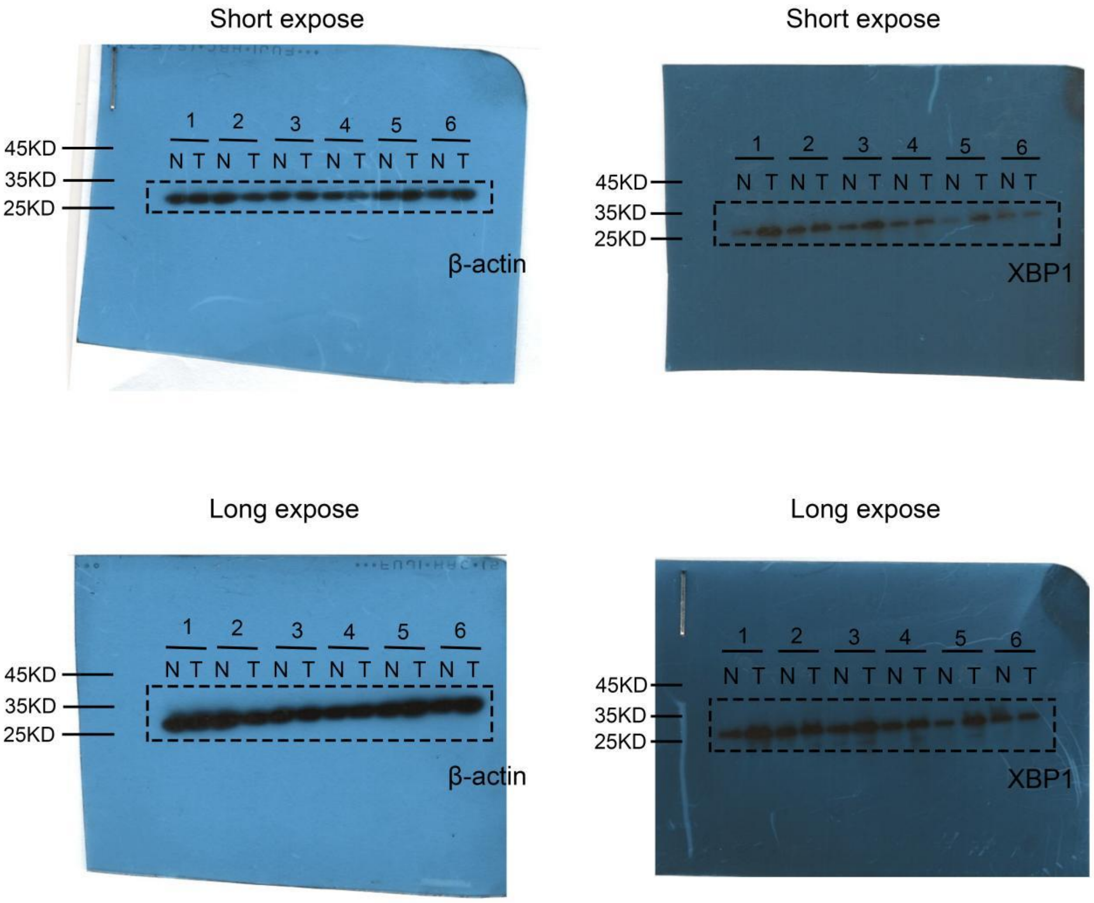

Supplementary Figure 4. Full unedited gel for Figure 9C.

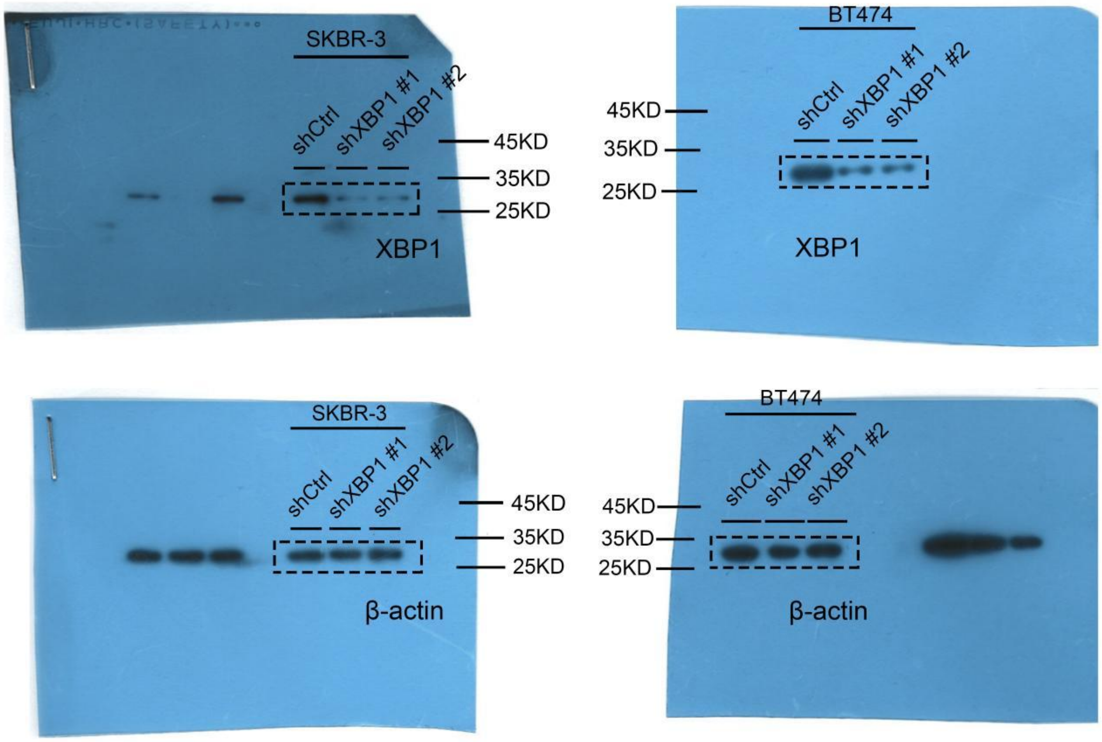

Supplementary Figure 5. Full unedited gel for Figure 10A.

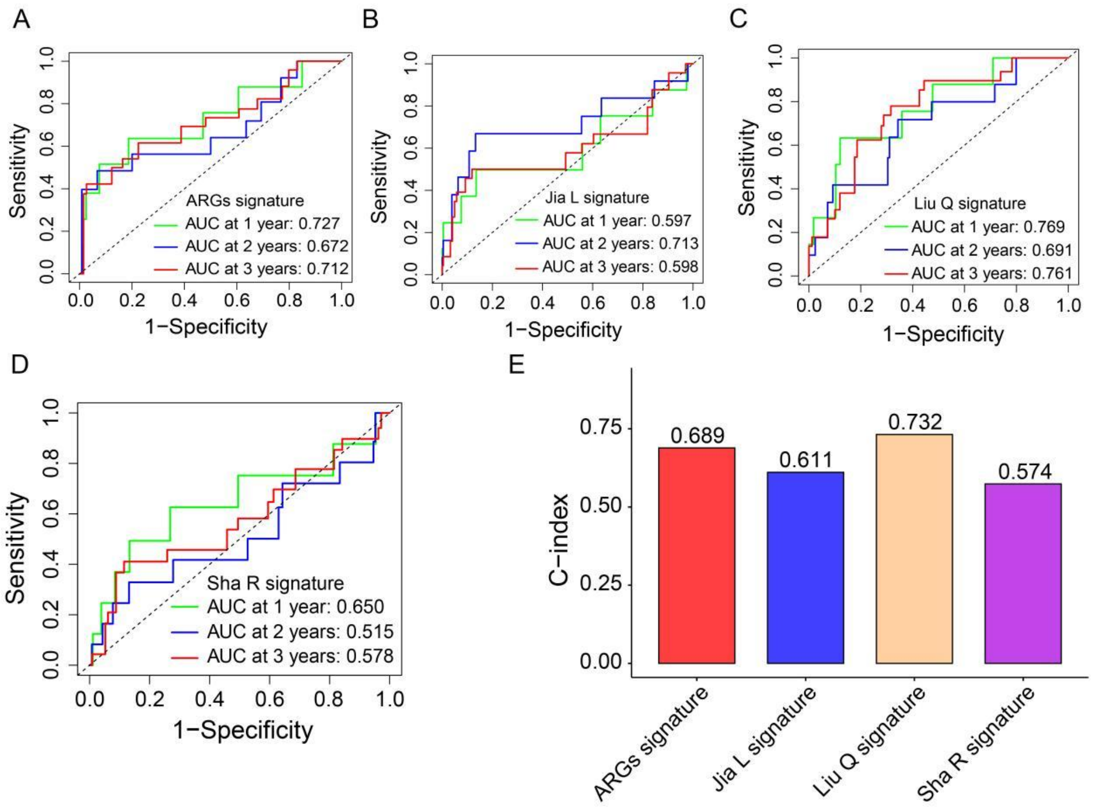

Supplementary Figure 6. Comparison between ARGs signature and 3 previously publish signatures. (**A**) Time-dependent receiver operating characteristic (ROC) curve of the ARGs prognostic signature. (**B**) ROC curve of the Jia L signature. (**C**) ROC curve of the Liu Q signature. (**D**) ROC curve of the Sha R signature. (**E**) C-index comparison of ARGs signature and 3 previously publish signatures in the TCGA cohort.
